# Reference chart for knee flexion following total knee arthroplasty: a novel tool for monitoring postoperative recovery

**DOI:** 10.1186/s12891-020-03493-x

**Published:** 2020-07-22

**Authors:** Andrew J. Kittelson, Jordi Elings, Kathryn Colborn, Thomas J. Hoogeboom, Jesse C. Christensen, Nico L. U. van Meeteren, Stef van Buuren, Jennifer E. Stevens-lapsley

**Affiliations:** 1 Physical Therapy Program, Department of Physical Medicine and Rehabilitation, University of Colorado, Aurora, CO USA; 2 School of Physical Therapy and Rehabilitation Science, University of Montana, Missoula, MT USA; 3 Department of Physiotherapy, University of Maastricht, Maastricht, The Netherlands; 4 Department of Biostatistics and Informatics, University of Colorado, Aurora, CO USA; 5 Radboud university medical Center, Radboud Institute for Health Sciences, IQ healthcare, Nijmegen, The Netherlands; 6 Department of Physical Therapy and Athletic Training, University of Utah, Salt Lake City, Utah USA; 7 VA Salt Lake City Health Care System, Salt Lake City, Utah USA; 8 Health~Holland (Topsector Life Sciences and Health), The Hague, The Netherlands; 9 Netherlands Organization for Applied Scientific Research TNO, Leiden, The Netherlands; 10 Department of Methodology and Statistics, University of Utrecht, Utrecht, The Netherlands; 11 Eastern Colorado VA Geriatric Research Education and Clinical Center (GRECC), Aurora, CO USA

**Keywords:** Generalized additive models for location scale and shape, Monitoring, Clinical decision making

## Abstract

**Background:**

Clinicians and patients lack an evidence-based framework by which to judge individual-level recovery following total knee arthroplasty (TKA) surgery, thus impeding personalized treatment approaches for this elective surgery. Our study aimed to develop and validate a reference chart for monitoring recovery of knee flexion following TKA surgery.

**Methods:**

Retrospective analysis of data collected in routine rehabilitation practice for patients following TKA surgery. Reference charts were constructed using Generalized Additive Models for Location Scale and Shape. Various models were compared using the Schwarz Bayesian Criterion, Mean Squared Error in 5-fold cross validation, and centile coverage (i.e. the percent of observed data represented below specified centiles). The performance of the reference chart was then validated against a test set of patients with later surgical dates, by examining the centile coverage and average bias (i.e. difference between observed and predicted values) in the test dataset.

**Results:**

A total of 1173 observations from 327 patients were used to develop a reference chart for knee flexion over the first 120 days following TKA. The best fitting model utilized a non-linear time trend, with smoothing splines for median and variance parameters. Additionally, optimization of the number of knots in smoothing splines and power transformation of time improved model fit. The reference chart performed adequately in a test set of 171 patients (377 observations), with accurate centile coverage and minimal average bias (< 3 degrees).

**Conclusion:**

A reference chart developed with clinically collected data offers a new approach to monitoring knee flexion following TKA.

## Background

Total knee arthroplasty (TKA) is one of the most common inpatient elective surgeries worldwide. There are approximately 700,000 procedures performed per year in the United States, and surgical rates are comparable in many European countries (120–200 per 100,000 people) [1, 2]. Despite the prevalence of TKA, there is little agreement in the clinical community regarding postoperative care and rehabilitation [3–5]. Rehabilitation practices vary widely by clinical site, and the content and goals of therapy are based largely on clinicians’ experience and intuition [3]. Postoperative protocols typically indicate recovery milestones based on the expected clinical course for the average person, but surgical populations are heterogeneous [6–8]. Fundamentally, clinicians and patients lack an evidence-based framework by which to judge individual patients’ recovery following TKA surgery, thus impeding efforts to advance personalized or patient-centered treatment approaches for this elective surgery.

Reference charts are commonly used in healthcare settings to assist in monitoring patients’ progress and to inform clinical decisions at the level of the individual patient. The concept of monitoring the clinical course and adapting treatment decisions according to observations at the individual level has been promoted in psychotherapy [9, 10], in patients with chronic disease [11], and more recently in research designs (one-person trials) [12]. In rehabilitation, reference charts have been proposed as a means of assessing patients’ response to preoperative inspiratory muscle training [13]. The key concept in all cases is to base clinical decisions on the comparison of individual-level observations to an evidence-based reference [14].

The goal of this study was to develop and validate a reference chart with clinically collected data, to inform monitoring of knee flexion active range of motion (AROM) following TKA surgery. Knee flexion is frequently assessed following TKA and is widely cited in postoperative protocols as a marker of progress throughout recovery [3, 15]. Additionally, we sought to describe a systematic approach to generating reference charts, including model fitting and selection procedures, so that future investigations could extend this methodology to other outcomes following TKA or to clinical trajectory data for other patient populations. Ultimately, this work could serve as a template for developing evidence-based references to aid in monitoring and decision-making for a variety of health conditions.

## Methods

Data were collected in the context of routine clinical practice at three sites (ATI Physical Therapy, in partnership with Greenville Health Systems) in South Carolina. As part of ongoing quality improvement efforts for patients undergoing knee arthroplasty, outcomes data were recorded on a semi-weekly basis throughout postoperative rehabilitation. Therapists were trained in the standardized application of outcomes assessments. The postoperative rehabilitation regime was also standardized across clinic locations and therapists. Outcomes data were compiled in a quality improvement (QI) database, housed at the University of Colorado Denver, using Research Electronic Data Capture (REDCap), a secure web-based software for database development. All analyses complied with a non-human subject research designation and were approved by the Colorado Multiple Institutional Review Board (COMIRB #: 15–1797).

### Patients

For the purposes of this analysis, the QI database was queried for all available (de-identified) patient records. At the time of data extraction, a total of 897 patient records were available, with surgical dates between January 2013 and May 2016. However, 321 records could not be used because they lacked postoperative flexion AROM data. This was not unexpected, as patients commonly travel to the surgical center for preoperative consultation and surgery but subsequently undergo postoperative rehabilitation at a clinic closer to home. An additional 59 records were excluded because patients underwent a procedure other than primary unilateral TKA (17 patients underwent revision arthroplasty and 42 patients underwent unicompartmental arthroplasty). For the remaining records, postoperative flexion AROM data were available between 2 and 857 days (median 36 days) following surgery. We utilized the first 120 postoperative days for this project. We reasoned that rehabilitation typically occurs during this time window, and recovery plateaus between months 1 and 3 following surgery [16]. Thus, we felt a reference chart describing recovery over the first 120 days would adequately capture the relevant time frame. A total of 498 patient records (1550 observations of flexion AROM) were used for this analysis.

### Knee flexion active range of motion (AROM)

Knee flexion AROM (in degrees) was measured in a supine position via long-arm goniometry (see supplementary material). Briefly, patients were allowed to practice bending their knee 5 times, with therapist-assist as needed, prior to the therapist making the final assessment. For the final assessment the knee was placed in extension, and the patient was instructed to flex the knee as far as possible using only muscle power, leaving the heel on the surface. The fulcrum of the goniometer was placed at the medial joint line, with the lateral malleolus of the fibula and greater trochanter of the femur as distal landmarks [17]. Physical Therapists were trained on a quarterly basis in this protocol, to standardize the collection of outcomes measures. Flexion AROM was measured on a semi-weekly basis throughout postoperative rehabilitation.

### Data analysis

We divided the sample of patient records temporally (by surgical date) into a development set and test set. By this approach, the development set contained the earlier 75% of the knee flexion measurements and the test set contained the later 25% of the available knee flexion measurements. Model fit was adjudicated and a reference chart was constructed using the development set, and the performance of this chart was subsequently examined using the test set. The steps for generating and assessing reference curves were analogous to those used to develop reference charts for childhood growth, emphasizing procedures that would also yield a simple solution that limits the risk of overfitting the data (Table 1) [18–20].

**Table 1:** Strategy for reference chart development

1. Generate flexion by time curves using GAMLSS with a variety of candidate distributions (e.g. Normal, Gamma, Box-Cox). 2. Determine whether the addition of smoothing splines to median, variance, skewness and kurtosis improve fit of the models, using the Schwarz Bayesian Criterion (SBC) as a numerical guide. 3. Optimize the number of knots of smoothing splines and power transformation of time using the find.hyper function. 4. Compare model fit for different candidate distributions using SBC and Mean Squared Error (MSE) by 5-fold cross validation. The best model minimizes these metrics. 5. Examine reference charts for each of the candidate distributions to determine the percentage of data captured below each of the specified centiles. The best model accurately represents the observed data (e.g. 5% below the 5th percentile, etc.). 6. For similar models, a less-complex approach (fewer degrees of freedom) is preferred.	

### Reference chart development

Using data from the development set, a series of statistical models were examined describing the variation of flexion AROM over the first 120 days following surgery. Generalized Additive Models for Location Scale and Shape (GAMLSS, version 4.4.0) [20] were used to obtain estimates of the median and other fitted centiles as smooth functions of the measurements in days. In GAMLSS, a variety of distributions can be used to fit the mean/median, variance, skewness, and kurtosis of the outcome. We selected 6 candidate distributions, of increasing complexity, for which to model knee flexion AROM. The Normal (NO) and Gamma (GA) distributions modeled 2 parameters (the median and variance) of the outcome. The t-family (TF) and Box-Cox Cole and Green (BCCG) distributions modeled the median, variance, and skewness of the outcome. The Box-Cox t distribution (BCT) and Box-Cox Power Exponential (BCPE) distributions modeled the median, variance, skewness and kurtosis of the outcome [21, 22]. Model fit was adjudicated numerically by the Schwarz Bayesian Criterion (SBC) [23]. To protect against over-fitting, we also calculated the Mean Square Error (MSE) via 5-fold cross validation of each model (i.e. by developing the model in 80% of the development set and testing the model in the left-out 20%). Based on these metrics, we pursued model selection by the following approach:

First, we examined whether fitting cubic splines for each of the different parameters (i.e. median, variance, skewness, kurtosis) improved model fit. Next, we optimized: 1) the number of knots specified in splines for each parameter, and 2) the power-transformation of time, using the “find.hyper” function in GAMLSS. We then constructed reference charts and calculated the percentage of observed values captured below each of the specified centiles (5th, 10th, 25th, 50th, 75th, 90th, and 95th centile). Of the candidate models, the best solution would minimize the SBC, demonstrate low MSE by within-sample cross validation, and accurately describe percentiles in the dataset, both within the development set as well as when applied to the test dataset (e.g. 5% of the observed data would be captured below the 5th percentile, 10% below the 10th percentile, etc.).

### Preliminary validation

Reference chart performance was examined by applying the reference curves to a test set of patients with later surgical dates. This approach was designed to mimic the process for development and subsequent use of the reference chart in practice. The accuracy with which the reference curves fit the new data was examined by z-test for proportions, and the average bias (difference between predicted and observed values) was calculated. Ideal performance would be reflected by accurate representation of the test set data (e.g., 5% of the observed data captured below the 5th percentile, 10% below the 10th percentile, etc.), and zero bias.

## Results

Demographic and anthropometric characteristics did not differ significanty between the development and test datasets (Table 2). The development set included 327 patients, with surgical dates between January 2013 and August 2015, while the test set included 177 patients with surgical dates between August 2015 and May 2016.

**Table 2:** Demographic and anthropometric characteristics of patients used to develop the reference chart (development set) vs. patients used to examine the performance of the reference chart (test set). Values are presented as mean ± standard deviation unless otherwise reported

	Development Set	Test Set	t-test (CHI^**2**^)
	***n*** = 327 (1173 obs)	***n*** = 171 (377 obs)	***p***-value
Age (yrs)	64.3 ± 9.4	64.9 ± 13.5	0.72
BMI (kg/m2)	32.9 ± 6.6	31.4 ± 7.9	0.23
Sex distribution (% female)	57.1	55.7	(0.8)

*Abbreviations*: *BMI* Body Mass Index, *obs* observations

### Model selection

For each of the 6 selected GAMLSS distributions (NO, GA, TF, BCCG, BCT, BCPE) we examined model fit under a variety of conditions. According to SBC, it was beneficial to model the location (mean/median) and variance parameters with cubic splines (Table 3). However, fitting splines to skewness and kurtosis parameters did not yield additional improvements in model fit (Table 3). Further improvements in SBC were achieved by optimizing the power transformation of time and the number of knots in smoothing splines (Table 4). Additionally, the process of optimization yielded simpler models with fewer overall degrees of freedom. The optimized models specified 2.2 knots for the median and 1.2 knots for variance, with a power transformation approximating the square root of time (0.56).

**Table 3:** Schwarz Bayesian Criterion (SBC) for models of increasing complexity (lower SBC values indicate a better solution), using data from the development set. Adding smoothing parameters for skewness and kurtosis parameters does not improve model fit

GAMLSS Distribution	Parameters with smoothing splines			
	Median	Median, Variance	Median, Variance, Skewness	Median, Variance, Skewness, Kurtosis
NO	9343.77	9309.87	–	–
GA	9503.95	9379.81	–	–
TF	9333.56	9313.83	9312.84	–
BCCG	9334.84	**9281.30**	9292.47	–
BCT	9341.22	9288.37	9299.56	9306.63
BCPE	9341.87	9285.50	9295.17	9301.96

*Abbreviations*: *NO* Normal, *GA* Gamma, *TF* t-Family, *BCCG* Box Cox Cole and Green, *BCT* Box Cox t-distribution, *BCPE* Box Cox Power Exponential

**Table 4:** Characteristics of GAMLSS models fit with smoothing splines for the median and variance, following optimization of smoothing spline knots and the power transformation of time. The best GAMLSS distribution for each metric is bolded. Results reflect within sample performance (i.e., within the development set)

GAMLSS Distribution	Model Degrees of Freedom	SBC	MSE	Percentage of observed values captured below model centiles				
				5th	25th	50th	75th	95th
NO	**7.45**	9298.6	**166.0**	6.05	23.53	47.49	73.49	97.1
GA	**7.45**	9364.7	165.6	6.05	22.59	45.69	73.83	98.04
TF	8.45	9303.0	171.1	6.05	24.47	48.34	73.49	97.19
BCCG	8.45	**9266.5**	166.4	**5.12**	**25.15**	**49.87**	74.51	95.14
BCT	9.45	9273.5	167.4	**5.12**	**25.15**	**49.87**	74.51	95.14
BCPE	9.45	9271.0	166.3	5.29	24.55	49.79	**74.85**	**95.06**

*Abbreviations*: *SBC* Schwarz Bayesian Criterion, *MSE* Mean Squared Error, *NO* Normal, *GA* Gamma, *TF* t-Family, *BCCG* Box Cox Cole and Green, *BCT* Box Cox t-distribution, *BCPE* Box Cox Power Exponential

**Fig. 1:**
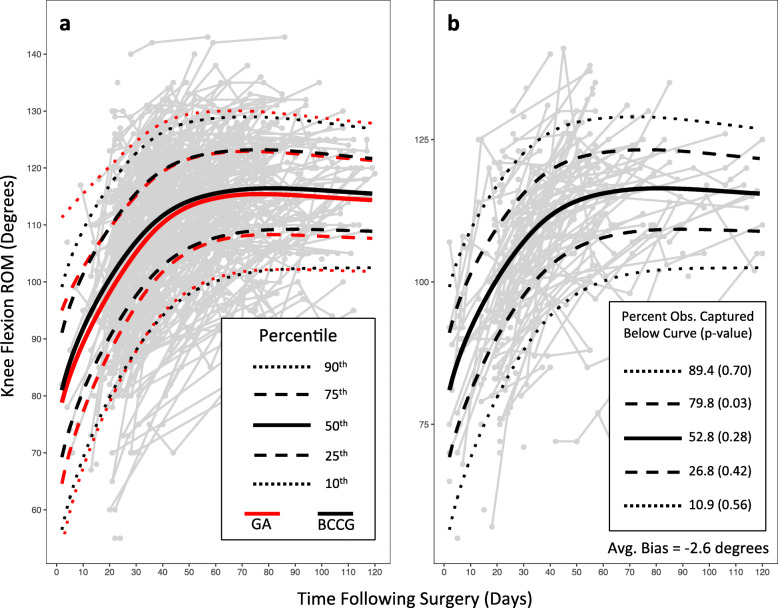
Knee flexion active range of motion (AROM) reference curves, applied to: **a** the development set (from which the curves were derived), and **b** the test set (a temporally distinct sample of patients). The worst fitting model (GA) and best fitting model (BCCG) according to Schwarz Bayesian Criterion are displayed (**a**). The BCCG model is applied to the test set (**b**), and the percent of observations captured below each of the specified centiles is provided. The *p*-value, according to general z-test, describes the probability of the observed percentage, given the expected percentage. The average bias describes the mean difference between observed values and values predicted from the GAMLSS model

Overall, reference charts for the optimized models demonstrated similar fit statistics and within-sample performance (i.e. within the development set). The BCCG, BCT, and BCPE distributions performed marginally better than the NO, GA, and TF distributions in representing percentiles of the development set data. For example, 45.7% of the observed data fell below the median for the reference chart built with a GA distribution, whereas 49.9% of the observed data fell below the median for the reference chart built with the BCCG distribution (Fig. 1a). The BCCG distribution performed incrementally better than BCPE in centile fit, and incrementally better than BCT according to SBC and MSE. Additionally, the BCCG model was the simpler model, with fewer overall degrees of freedom. Based on these metrics we chose to build the final reference chart for flexion AROM using the BCCG distribution.

### Preliminary validation

The performance of the curves in the test set was slightly diminished relative to the performance in the development set. For example, 80% of the observations fell below the 75th percentile (*p* = 0.03 for the difference between the observed and expected proportions). However, other observed proportions were similar to expected proportions, and the average bias remained minimal at − 2.5 degrees of flexion AROM (Fig. 1b).

### Reference values and chart

**Fig. 2:**
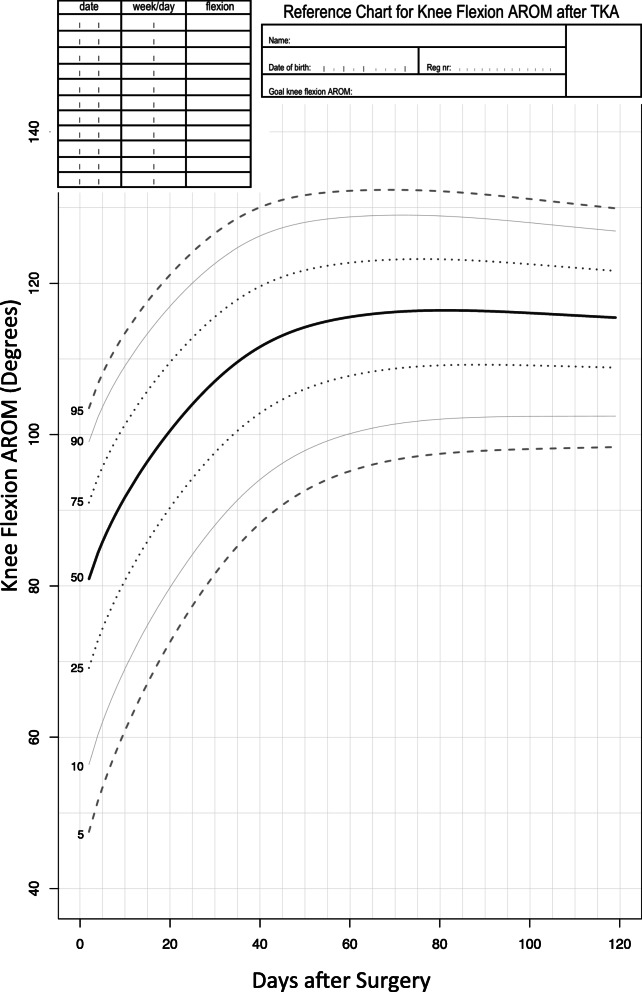
Reference chart for monitoring knee flexion range of motion (AROM) following TKA surgery

Figure 2 shows the reference chart we developed for monitoring flexion AROM after TKA. It gives a sense of the general trajectory and variability in postoperative recovery of flexion AROM over the first 120 days following surgery. The typical recovery trajectory demonstrates flexion AROM between 70 and 90 degrees (interquartile range) immediately following surgery, 95–115 degrees 1 month following surgery, and 109–122 degrees 3 months following surgery.

## Discussion

This reference chart illustrates how flexion AROM changes following surgery. In general, there is a period of rapid increase within the first 40 days, followed by a gradual plateau. The shape of the recovery trajectory differs depending on the centile of the reference chart. Higher centiles plateau more rapidly than lower centiles, with the lowest centiles continuing to demonstrate improvements up until 60–80 days following surgery. Moreover, there is substantial variability between individual patients in recovery of knee flexion. The interquartile range is between 13 and 22 degrees throughout the first 4 months of recovery. This variability illustrates the limitations of a one-size-fits-all approach to postoperative rehabilitation, as both the content of therapy and resource requirements are likely to differ between individuals with fast versus slow recovery of flexion. Tools such as this reference chart extend the work of previous studies (which have modeled the trajectory of recovery of the sample mean) [16, 24] to include an intuitive display of the variability in recovery, which may facilitate decisions at the level of the individual patient [25].

This flexion AROM reference chart represents a departure from one-size-fits-all protocols, which have traditionally been used to guide decisions following TKA surgery [15]. A typical TKA rehabilitation protocol might stipulate a postoperative flexion AROM goal of 110 degrees, as this is the amount of knee flexion required for many activities such as symmetrical stair gait or cycling [26]. However, at an individual level, patients may have more or less ambitious functional goals, and this reference chart provides an indication of the likelihood (as well as the timing) of whatever is best for the individual. Moreover, the variability in knee flexion AROM outcomes, readily apparent on the reference chart, further illustrates the problems in stipulating a single goal for all patients. According to the reference chart, a patient who demonstrates > 90 degrees of flexion AROM early after surgery should ultimately achieve an outcome much greater than 110 degrees, whereas a patient who demonstrates < 60 degrees of flexion AROM early after surgery has a high likelihood of not achieving 110 degrees. Thus, the reference chart accommodates a more nuanced picture of the clinical course, which could augment or replace protocol-based approaches to rehabilitation [14].

A strength of our study is the use of data collected in routine clinical practice, as the results are less likely to be influenced by volunteer bias or research eligibility criteria. This potentially enhances the future translation to rehabilitation clinical settings. The temporal validation used in this study is also a strength; it suggests the reference chart remains accurate over time. However, as our data were collected in a single clinic system (3 sites), the geographic generalizability is unknown. The references reported here could be compared to data collected at other sites in future work. Another potential limitation is the time frame selected for chart development. We limited our dataset to the first 4 postoperative months to cover the typical time frame of rehabilitation, but recovery may persist at a slower rate for many additional months [27]. Thus, there may be value in expanding this reference chart to cover a longer post-operative time frame. Finally, the reference chart presented here describes knee flexion, but multiple outcome measures are likely to be important to individuals and clinicians following TKA surgery [28, 29]. Future work could focus on developing a menu of reference charts describing a range of clinical outcome measures (e.g. physical functioning, pain, quality of life) to support recovery monitoring [14].

## Conclusion

We have developed and tested a reference chart for knee flexion AROM following TKA surgery. The final reference chart was accurate via both within-sample and out-of sample testing. It is designed to be easy to use in practice to track postoperative recovery of knee flexion AROM for individual patients, relative to others who have previously undergone TKA surgery.

## Supplementary information

**Additional file 1.** ICMJE Form for Disclosure of Potential Conflicts of Interest. PDF files contain all the ICMJE Form for Disclosure of Potential Conflicts of Interest for each author.

## Data Availability

The datasets used for this analysis may be available upon reasonable request. Please contact the senior author, Dr. Jennifer Stevens-Lapsley, for details.

## Abbreviations

TKA: Total knee arthroplasty
CDC: Centers for Disease Control
WHO: World Health Organization
AROM: Active range of motion
QI: Quality improvement
REDCap: Research Electronic Data Capture
GAMLSS: Generalized Additive Models for Location Scale and Shape
TF: T-family
NO: Normal
GA: Gamma
BCCG: Box-Cox Cole and Green
BCT: Box-Cox t distribution
BCPE: Box-Cox Power Exponential
SBC: Schwarz Bayesian Criterion
MSE: Mean Square Error

## References

[1] Facts and Figures 2009 (2017). Healthcare Cost and Utilization Project (HCUP).

[2] Kurtz SM, Ong KL, Lau E, Widmer M, Maravic M, Gomez-Barrena E (2011). International survey of primary and revision total knee replacement. Int Orthop.

[3] Peter WF, Nelissen RG, Vlieland TP (2014). Guideline recommendations for post-acute postoperative physiotherapy in total hip and knee arthroplasty: are they used in daily clinical practice?. Musculoskeletal Care.

[4] NIH Consensus Statement on total knee replacement (2003). NIH consensus and state-of-the-science statements.

[5] Naylor JM, Hart A, Harris IA, Lewin AM (2019). Variation in rehabilitation setting after uncomplicated total knee or hip arthroplasty: a call for evidence-based guidelines. BMC Musculoskelet Disord.

[6] Weiss JM, Noble PC, Conditt MA, Kohl HW, Roberts S, Cook KF (2002). What functional activities are important to patients with knee replacements?. Clin Orthop Relat Res.

[7] Naylor JM, Harmer AR, Heard RC, Harris IA (2009). Patterns of recovery following knee and hip replacement in an Australian cohort. Aust Health Rev.

[8] Lugano G, Gianola S, Castellini G, Banfi G, Seil R, Denti M (2020). Evidence-based medicine (EBM) is properly perceived but its application is still limited in the orthopedic clinical practice: an online survey among the European Society of Sports Traumatology, knee surgery and arthroscopy (ESSKA) members. Knee Surg Sports Traumatol Arthrosc.

[9] Lutz W, Rafaeli E, Howard KI, Martinovich Z (2002). Adaptive modeling of progress in outpatient psychotherapy. Psychother Res.

[10] Lueger RJ, Howard KI, Martinovich Z, Lutz W, Anderson EE, Grissom G (2001). Assessing treatment progress of individual patients using expected treatment response models. J Consult Clin Psychol.

[11] Glasziou P, Irwig L, Mant D (2005). Monitoring in chronic disease: a rational approach. Brit Med J.

[12] Schork NJ (2015). Personalized medicine: time for one-person trials. Nature.

[13] van Buuren S, Hulzebos EH, Valkenet K, Lindeman E, van Meeteren NL (2014). Reference chart of inspiratory muscle strength: a new tool to monitor the effect of pre-operative training. Physiotherapy.

[14] Kittelson AJ, Hoogeboom TJ, Schenkman M, Stevens-Lapsley JE, van Meeteren NLU (2020). Person-centered care and physical therapy: a “people-like-me” approach. Phys Ther.

[15] Kisner C, Colby LA (2012). Therapeutic exercise: foundations and techniques.

[16] Kennedy DM, Stratford PW, Riddle DL, Hanna SE, Gollish JD (2008). Assessing recovery and establishing prognosis following total knee arthroplasty. Phys Ther.

[17] Brosseau L, Balmer S, Tousignant M, O'Sullivan JP, Goudreault C, Goudreault M (2001). Intra- and intertester reliability and criterion validity of the parallelogram and universal goniometers for measuring maximum active knee flexion and extension of patients with knee restrictions. Arch Phys Med Rehabil.

[18] Hayes DJ, van Buuren S, ter Kuile FO, Stasinopoulos DM, Rigby RA, Terlouw DJ (2015). Developing regional weight-for-age growth references for malaria-endemic countries to optimize age-based dosing of antimalarials. Bull World Health Organ.

[19] Flegal KM, Cole TJ (2013). Construction of LMS parameters for the Centers for Disease Control and Prevention 2000 Growth charts. Nat Health Stat Rep.

[20] Stasinopoulos DM, Rigby RA, Akantziliotou C (2008). Instructions on how to use the gamlss package in R.

[21] Rigby RA, Stasinopoulos DM (2004). Smooth centile curves for skew and kurtotic data modelled using the box-cox power exponential distribution. Stat Med.

[22] Rigby RA, Stasinopoulos DM (2006). Using the box-cox t distribution in GAMLSS to model skewness and kurtosis. Stat Model.

[23] Cavanaugh JE, Neath AA (1999). Generalizing the derivation of the schwarz information criterion. Commun Stat Theory Methods.

[24] Kennedy DM, Stratford PW, Hanna SE, Wessel J, Gollish JD (2006). Modeling early recovery of physical function following hip and knee arthroplasty. BMC Musculoskelet Disord.

[25] Kraemer HC, Frank E, Kupfer DJ (2006). Moderators of treatment outcomes: clinical, research, and policy importance. JAMA.

[26] Rowe PJ, Myles CM, Walker C, Nutton R (2000). Knee joint kinematics in gait and other functional activities measured using flexible electrogoniometry: how much knee motion is sufficient for normal daily life?. Gait Posture.

[27] Bade MJ, Kittelson JM, Kohrt WM, Stevens-Lapsley JE (2014). Predicting functional performance and range of motion outcomes after total knee arthroplasty. Am J Phys Med Rehabil.

[28] Yoshida Y, Mizner RL, Ramsey DK, Snyder-Mackler L (2008). Examining outcomes from total knee arthroplasty and the relationship between quadriceps strength and knee function over time. Clin Biomech.

[29] Mizner RL, Petterson SC, Clements KE, Zeni JA, Irrgang JJ, Snyder-Mackler L (2011). Measuring functional improvement after total knee arthroplasty requires both performance-based and patient-report assessments: a longitudinal analysis of outcomes. J Arthroplast.

